# A Framework to Optimize Primary Care of Older Surgical Patients: A Qualitative Study of Geriatricians

**DOI:** 10.1001/jamanetworkopen.2024.56787

**Published:** 2025-01-27

**Authors:** Molly E. Leonard, Andrea J. H. Williamson, Roxanne Weiss, Kimberly A. Kaphingst, Mark A. Supiano, Jessica N. Cohan

**Affiliations:** 1 Division of General Surgery, University of Utah, Salt Lake City; 2 Division of Geriatrics, University of Utah, Salt Lake City; 3 Department of Communications, University of Utah, Salt Lake City; 4 Huntsman Cancer Institute, Salt Lake City, Utah; 5 Division of General Internal Medicine, University of Utah, Salt Lake City; 6 Division of Surgical Oncology, University of Utah, Salt Lake City

## Abstract

**Importance:**

An increasing number of older adults are undergoing surgery. Older adults face significant challenges throughout the spectrum of perioperative care. No frameworks exist to support primary care clinicians in helping older adults navigate perioperative care beyond preoperative medical clearance.

**Objective:**

We aimed to develop a framework to assist primary care clinicians in surgical care navigation for older patients.

**Design, Setting, and Participants:**

This qualitative study used semistructured interviews with a sample of geriatricians from across the US between January and June 2022. Interviews were conducted one on one via an online video conferencing platform without observers present. Demographics of participants were analyzed using descriptive statistics.

**Main Outcome and Measures:**

Directed content analysis of interview data was used to examine how primary care clinicians can optimally support older patients throughout the perioperative process.

**Results:**

This qualitative study included 24 geriatricians, 16 (67%) women, with median time in practice of 12.4 years (IQR, 5.0-24.5 years). Of those, 11 (46%) worked at an academic or tertiary referral center. Qualitative analysis identified 7 actions that geriatricians perform when caring for patients through the surgical continuum: conduct risk-benefit analysis of surgical referral, elicit and communicate patient goals, prepare patient and family for surgical consultation, set realistic expectations, assist with decision about surgery, advocate for patient and family, and coordinate postoperative care.

**Conclusions and Relevance:**

In this qualitative study of geriatricians, 7 key domains of perioperative care for older adults were identified. The resulting framework can be used by primary care clinicians as they help their older patients navigate surgical care.

## Key Points

**Question:**

How can older adults be optimally supported during the surgical care process by primary care clinicians?

**Findings:**

This qualitative study of semistructured interviews with 24 geriatricians identified 7 actions that geriatricians undertake in addition to routine preoperative optimization: conduct risk-benefit analysis of surgical referral, elicit and communicate patient goals, prepare patient and family for surgical consultation, set realistic expectations, assist with decision about surgery, advocate for patient and family, and coordinate postoperative care.

**Meaning:**

Our findings establish a comprehensive framework that primary care clinicians may reference when caring for older patients who may require elective surgery.

## Teaser

This qualitative study presents a framework to assist primary care clinicians in navigating surgical care for older patients.

## Introduction

The population of the United States is aging, with the number of adults older than 65 years expected to nearly double to 95 million people by 2060.^1^ Caring for older adults is complex and requires managing chronic conditions, polypharmacy, cognitive impairment and frailty, and attention to psychosocial situations and goals of care.^2^ Internal and family medicine physicians provide the majority of primary care for older adults and are expected to do so for the foreseeable future.^3,4^ Although several programs for internal medicine residents and physicians have been developed to enhance geriatric education,^5,6,7^ there is no national standard. The result is that primary care clinicians receive little formalized training in geriatrics, especially regarding perioperative care.

Older adults are undergoing an increasing number of surgical procedures.^8^ Improving surgical care for older adults has become an increasing focus within the surgical community as evidenced by multiple geriatric quality initiatives, such as the American College of Surgeon’s Geriatric Surgery Verification Program and the Institute for Healthcare Improvement’s Age Friendly Healthcare Systems movement.^9,10^ Optimizing perioperative care for the patients is also becoming increasingly important from a hospital administration perspective, as, for example, the Centers for Medicare & Medicaid services are considering a geriatric surgery measure for the Hospital Inpatient Quality Reporting Program.^11^

Guidelines exist regarding preoperative patient optimization, medical clearance, and inpatient postoperative medical care.^9,12,13^ However, there is minimal guidance for how primary care clinicians can help their older patients navigate surgical care in the preoperative and postoperative settings. Providing primary care clinicians with evidence-based insight into how to assist with decision-making about pursuing surgery, communicate with the surgical team, advocate for their patients, and support transitions of care will help optimize surgical care for older adults. We performed a qualitative study to understand the experience and practice patterns of geriatricians whose patients require surgery. We explored their perspectives on the perioperative care process, from the time that a patient presents with a surgical disease through their surgical recovery. This information was used to develop a framework to optimize primary care delivery for older patients requiring surgery.

## Methods

### Study Design

This qualitative study used semistructured interviews with a national sample of geriatricians to develop a framework to assist primary care clinicians in surgical care navigation for older patients. This study was designed and conducted according to the Consolidated Criteria for Reporting Qualitative Research (COREQ) reporting guideline.

We used a convenience sampling method to recruit geriatricians across the US by posting on the online American Geriatrics Society Member Forum. Given this recruitment method, it is unknown how many potential participants were reached. A total of 2 advertisements were posted 3 weeks apart. Interested parties contacted the study team and underwent screening for eligibility. We excluded those who primarily practiced in inpatient and nursing home settings because the purpose of this study was to understand the experience of geriatricians who provide primary care. Eligible participants were offered a $50 incentive for participating.

This study was approved by the University of Utah institutional review board. All participants received a consent cover letter and provided verbal consent before starting the interview.

Demographic race and ethnicity data were self-reported by participants via an online questionnaire using REDCap (Research Electronic Data Capture). A semistructured interview guide was developed to elicit geriatrician perspectives on caring for older patients with surgical disease. The development team consisted of one surgeon (J.N.C.) with input from a geriatrician (M.A.S.) and an expert in qualitative research (K.A.K). The interview script had 2 main purposes. First, we aimed to understand geriatrician practices in providing care to older patients navigating the surgical process. Second, we sought to examine geriatricians’ perspectives on surgeon-geriatrician interactions and how surgeons can partner with geriatricians to improve care. Findings from the second analysis will be reported separately. The interview script (eAppendix in Supplement 1) was pilot tested with a board-certified geriatrician.

Interviews were conducted by A.J.H.W., a female surgical resident, training in qualitative methods and experience conducting interviews for qualitative research. She had no preexisting relationship with participants. Prior to the interview, her role as a surgical resident and her interest in optimizing surgical care for older adults was revealed to the participants.

Interviews were conducted one on one via an online video conferencing platform without observers present. The interview script was not made available to the participants. No field notes were made during the interviews. Interviews were conducted between January and June 2022, when thematic saturation was reached. No interviews were repeated. The mean length of the interviews was 32 minutes 58 seconds (±5 minutes, 56 seconds). After completion, audio recordings of the interviews were professionally transcribed verbatim. Participants were not given the opportunity to review interview transcripts.

### Statistical Analysis

Demographics of participants were analyzed using descriptive statistics. Data analysis was conducted from June 30, 2022, to September 9, 2022. Four members of the study team (A.J.H.W., M.E.L., J.N.C., and R.W.) carried out qualitative coding and analysis of the interview transcripts. This was overseen by an expert in qualitative methods (K.A.K.). We used a conventional content analysis approach, drafting the initial codebook based on major preliminary themes.^14^ Codebook revision took place through an iterative process involving all study team members. First, 3 transcripts (12.5%) were coded by all team members to guide codebook clarification and modification. After this, all transcripts were coded and in subsequent rounds of coding, 20% of transcripts were co-coded. Discrepancies in codes for transcripts that were co-coded were resolved through consensus of team members. The final codebook pertaining to this analysis of geriatrician practices in supporting patients through the perioperative process included 7 main codes and 20 subcodes. Analysis of codes took place through an inductive process. Results were not shared with participants for feedback. Coding was performed using Microsoft Excel, version 16.89.

## Results

### Participant Demographics

Twenty-eight geriatricians expressed interest in participating in the study. Four were lost to follow-up. Of the 24 geriatricians (16 [67%] women) who enrolled and were interviewed, the median time in practice was 12.4 years (IQR, 5.0-24.5 years), and 11 (46%) worked at an academic or tertiary referral center. Characteristics of study participants are shown in Table 1.

**Table 1.** Characteristics of the Study Population

Characteristic	Participants, No. (%) (N = 24)
Age, mean (IQR), y	46.5 (37-54)
Years in practice, mean (IQR)	12.5 (5.0-24.5)
No. of surgical referrals per month	4.5 (2-5)
Race	
Asian	9 (37.5)
Black or African American	1 (4.2)
White	13 (54.2)
>1 Category	1 (4.2)
Ethnicity	
Hispanic or Latinx	0
Not Hispanic of Latinx	24 (100)
Gender	
Woman	16 (66.7)
Man	8 (33.3)
Degree	
Physician (MD, DO, MBBS)	23 (95.8)
Advanced practice clinician (PA, NP)	1 (4.2)
Specialty within geriatrics outside of primary care	
Yes	6 (25.0)
No	18 (75.0)
Practice type	
Academic or tertiary care	11 (45.8)
Hospital employed or community	6 (25)
Private or group practice	4 (16.7)
Veterans Affairs	1 (4.2)
PACE Program	2 (8.3)
Region	
West	7 (29.2)
Midwest	3 (12.5)
Northeast	7 (29.2)
South	7 (29.2)
Time spent on outpatient care, %	
0-25	4 (16.7)
26-50	3 (12.5)
51-75	4 (16.7)
76-100	13 (54.2)
Time spent on primary care, %	
0-25	5 (20.8)
26-50	2 (8.3)
51-75	3 (12.5)
76-100	14 (58.3)

Abbreviations: DO, doctor of osteopathic medicine; MBBS, bachelor of medicine, bachelor of surgery; MD, doctor of medicine; NP, nurse practitioner; PA, physician assistant; PACE, Program of All-Inclusive Care for the Elderly.

From the thematic analysis, 7 overarching themes emerged representing actions performed by primary care geriatricians to support patients through the perioperative process in addition to typical preoperative clearance. The major themes identified were conducting a risk-benefit analysis of the surgical referral, eliciting and communicating patient goals, preparing the patient and family for surgical consultation, setting realistic expectations, assisting with decision about surgery, advocating for the patient and family, and coordinating postoperative care. These actions began when a patient was identified to have a disease that may require surgery and many continued through the perioperative period until surgical recovery. We organized these actions into a framework that primary care clinicians may follow when guiding older patients through the time of a surgical diagnosis through postoperative recovery (Figure).

**Figure.**
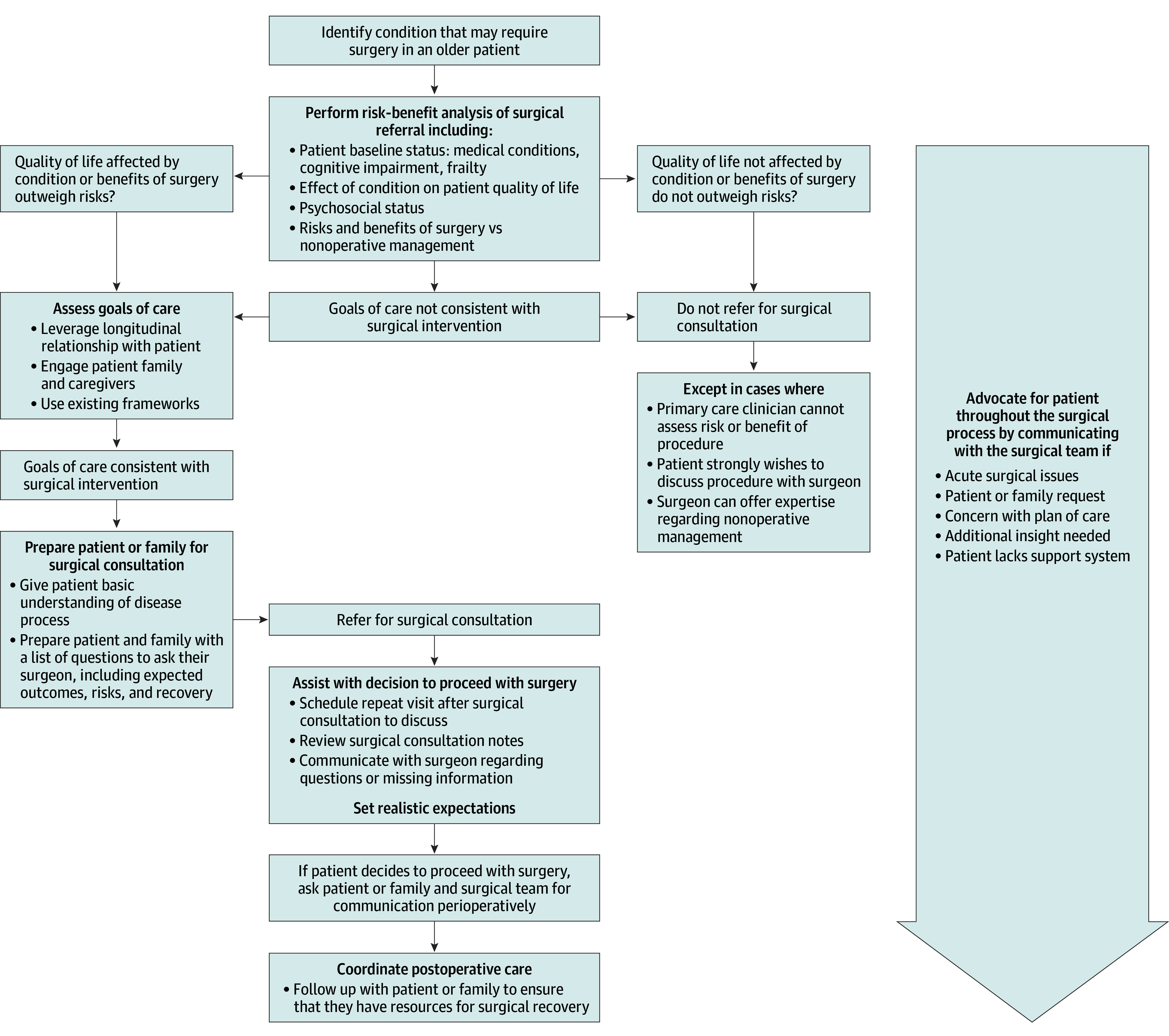
Framework for Primary Care Clinicians to Support Older Patients With Surgical Conditions The framework is based on geriatrician actions that primary care clinicians may use when supporting older patients throughout the surgical continuum.

#### Risk-Benefit Analysis of Surgical Referral

When deciding whether to refer a patient for a surgical consultation, geriatricians perform a comprehensive, multidimensional assessment of their patient, including an evaluation of the patient’s baseline medical, cognitive status, functional status, quality of life, and frailty. Geriatricians then perform a risk-benefit analysis comparing surgery to nonoperative management. The synthesis of this analysis is used to determine whether to refer a patient to a surgeon (Table 2).

**Table 2.** Factors That Geriatricians Consider in Their Risk-Benefit Analysis When Deciding Whether to Refer a Patient for Surgical Consultation

Factor	Core meaning	Exemplar quotes
Patient baseline status	Evaluate medical conditions, cognition, frailty, and functional status.	“I like to assess their pre-op risk, which means I look at their medical conditions, their comorbidities, especially their medications and if there’s any potential pre-op medication adjustments, I might have to do like blood thinners or blood pressure medications or anything like that, and I also like to look at their cognitive function. So, with frailty I start to worry about if they’re at increased risk for delirium or if they have existing cognitive issues like dementia too, so I think that would guide my discussions on whether it’s worth doing a particular procedure. Then function-wise, like ADL’s and IDL’s or mobility, balance and falls and that kind of thing. If let’s say they’re really frail and they have difficulty walking already, what’s the chance that they’re going to need to go to a nursing home post-op, and how is the discharge planning going to be affected by that.” (Participant 10)
Impact of surgical condition on quality of life	Evaluate the extent to which the surgical condition is affecting the patient’s quality of life.	“You have to consider the risk and benefits of any surgery in light of their individual frail conditions, how much discomfort is this hernia causing, how much discomfort is this bad shoulder causing, and how much interference with their function do each of those cause, and if it appears that they have significant discomfort or significant reversible functional decline.” (Participant 2)
Risks and benefits of surgery	Is it likely that the patient will benefit from surgery?	“I guess G-tube and PEG tubes are … an example where you may not recommend it… and I’ve had patients or families who really want a procedure for whatever reason, and there’s clear evidence that doing that would be more harm.” (Participant 10)
Consider nonoperative management strategies	Are there nonsurgical options? If so, what are the risks and benefits?	“…This patient of mine who had multiple abdominal surgeries a couple of years ago… had an abdominal hernia and (it) was cosmetically bothersome. So, we had numerous conversations on how—what if we referred the person to a surgeon and there is a surgery, what would be the pros and cons of it and if this thing isn’t bothering the person. While this could eventually at some point be bothersome, but… become a medical emergency.” (Participant 26) “Let’s say we’re talking about a frail patient, and based on my pre-operative risk evaluation, he is at risk, then I would try… some symptomatic treatments to see if that helps and discuss the red flags.” (Participant 25)

Abbreviations: ADL, activities of daily living; IDL, instrumental activities of daily living; PEG, percutaneous endoscopic gastrostomy.

After completing this comprehensive patient assessment, geriatricians indicate that there are additional factors that lower the threshold for surgical referral. Some geriatricians feel that surgeons are more able to provide an accurate risk-benefit assessment for the patient:

I have a pretty low threshold to refer to general surgery, because the surgeon has to do their own risk assessment and their own counseling, which is more in-depth than my counseling. The surgeon can speak much more precisely about the pros and the cons. (Participant 9)

Other geriatricians also refer to surgeons for their input on nonoperative management of a condition:

Even if a person I didn’t think would benefit from surgery, I think… having the person go see the surgeon for other suggestions can still be helpful. (Participant 6).

#### Elicit and Communicate Patient Goals

Eliciting and communicating patient goals is a key action geriatricians perform when navigating a patient through the surgical process, guiding appropriate decision-making and clinical care:

Goals and preferences are part and parcel of good geriatric practice because these are patients often with limited life expectancy, and their goals may not always be my goals, and if I don’t know what they are we can’t align properly. (Participant 2)

Geriatricians identified 4 approaches to eliciting patient goals of care: leveraging longitudinal relationships with their patients, discussing current quality of life, using existing frameworks, and ensuring that caregivers and/or family members are included in the conversation when possible (Table 3). Further, geriatricians try to communicate goals of care in a way that makes them accessible to surgeons at the time of consultation:

When I send our personalized message to surgeon besides my note I really highlight [goals and preferences], so it’s… important, and I believe that has helped my surgeon colleagues to plan or counsel appropriately. (Participant 22)

However, they identify that this is often difficult to do in the electronic medical record (EMR):

**Table 3.** Approaches to Eliciting Patient Goals of Care When Considering Surgery

Approach	Exemplar quotes
Leverage longitudinal relationships	“So… this is an ongoing conversation for me. This is not just the one-time conversation because I had a relationship with my patients.” (Participant 24)
Discuss current quality of life	“How I start the conversation with my patients is ‘What is your quality of life now? And how it may improve, and how it might deteriorate?’” (Participant 24)
Use existing framework	“I always have a conversation about advance directives. I usually use the MOLST, which is the Maryland Medical Orders for Life-Seeking Treatment, as my kind of anchor.” (Participant 9)
Include family and caregivers in conversation	“Whenever possible, if there are close family members or people who help the person in decision-making, you know, even if they’re totally cognitively intact, it’s still a major decision, to talk about.” (Participant 1)

I hope there will be [a] more discrete place for all of those to be easily seen in the EMR, like if… the unique things about the patients will pop up so clearly that no one can ignore or skip their goals of care, what their values are or… what matters to them. (Participant 22)

#### Prepare Patient and Family for Surgical Consultation

After deciding to refer a patient for a surgical consultation, geriatricians help patients and families get ready for the consultation. This process includes giving patients education on the disease process that they are going to be seen for:

I’ll make sure they understand… their disease process, what are they undergoing, and I’ll understand some of them still have questions. We try to answer it as much as possible. (Participant 17)

Participants also describe coaching patients on questions that they or their support person may want to ask during their visit:

I try to set up the patient, I say this is the question you’re going to ask, these are the things that you’re going to discuss, and I try to repeat that as many times or talk to the caregiver. (Participant 10)

#### Set Realistic Expectations

Another role for geriatricians is ensuring that patients and their family members have accurate expectations about the outcomes from surgery:

A lot of patients… have very unrealistic expectations that they’re gonna be cured by surgery, so… if I talk to a patient about a total knee I’m gonna be very frank and say “Your pre-morbid level of function is not great. You’re gonna have at least a year recovery” and… gauge what they want to do, and some may choose not to do anything, and some say “You know, I’m in such pain, I can’t walk, I’m willing to take that risk,” and I’m willing to go that route with them, so I… try to see if their expectations are realistic. (Participant 23)

Geriatricians emphasize the importance of patients understanding the risks of surgery. They indicate concerns that this topic may not be adequately discussed during the surgical consultation:

My main concern, especially when they go to a surgeon, and I’m not convinced they need surgery, is that the surgeon will tell them, “Yes, I can do the surgery, and yes, it’ll be easy,” but the surgeon’s never going to tell them that they might have a delirium, might never go home again, might end up in a nursing home, or that maybe the pain won’t go away, because it’s not actually from that. (Participant 7)

Geriatricians also seek to ensure understanding of risks and outcomes from pursuing nonsurgical management:

We talk about whether we have an understanding regarding possible outcomes and consequences relating to going through with the procedure or delaying it. And I have the patient… talk to me about what their understanding is. (Participant 27)

#### Assist With Decision About Surgery

After the surgical consultation, participants frequently help the patient and family decide whether to proceed with surgery:

Once they have the… surgical consultation, I always read the note and then sometimes patients will come back to me and be like, “Well I want to talk to you and decide whether to have it.” And then I’ll try to use a best-case worst-case scenario… to… help figure out expectations and then I’ll also talk to the surgeon… to… make sure we’re on the same page. (Participant 20)

#### Advocate for Patient and Family

Geriatricians identify advocating for the patient and family throughout the perioperative process as an important part of their role:

I have to fight for the patient. I’m their advocate, and maybe the patient can’t speak up for themselves, or maybe a patient’s family member is timid or won’t talk to the surgeon or won’t ask the right questions or don’t know what questions to ask, so I’ll ask for them. (Participant 4)

Multiple situations that would require geriatrician advocacy for their patients were identified (Table 4).

**Table 4.** Reasons That Geriatricians Advocate for Patient and Family With Exemplar Quotes

Reason for geriatrician advocacy	Exemplar quotes
Acute surgical issue	“If it’s something that I need worked in faster than they can do, then I’ll call the surgeon directly.” (Participant 2)
Concern with plan of care	“If there is a particular concern I have about this patient going through with a particular surgery, then I will contact the surgeon by phone.” (Participant 27)
Provide additional insight	“I’ll try to shed light on any instincts that I have about what might be an issue with moving forward or any barriers that I could envision that, as a primary care doctor, I might have a better insight about, you know, having a longer-lasting relationship with the patient leading up to that event.” (Participant 9)
Patient or Family request	“So, sometimes if it is like a really complex situation or there’s a family member who’s a caregiver for the patient and is closely involved. Sometimes they’re MyCharting me every step along the way. And we’re communicating about things and they’re worried about things and they want me to weigh in on, ‘Oh, my god, she’s still in the hospital and she hasn’t been able to eat in three days and the team just comes in and out and I don’t know what’s going on.’ So, there are those situations where I then do my best to sort of say, ‘Okay, I’m looking at the hospital notes. Looks like your mom has an ileus, this is what they’re doing for it. Talk to the nurse… or talk to the doctor and ask to see the surgeon and ask these questions.’” (Participant 1)
Patient lacks other support system	“I do have a few patients that want me to be there with them either advocating because they don’t have family—they feel like they need that strong presence.” (Participant 4)

#### Postoperative Care Coordination

Geriatricians help prepare the patient and family for surgical recovery by discussing discharge disposition, patient social support, and other resources that may be needed:

Who they will need to have at home… what kind of services they might need. Would they need to go to a rehab? …If they need to call their family, or they need to hire an aide, or they to have somebody there to give them a shower, …whatever they might need, we’ve already discussed that on that visit. (Participant 24)

After leaving the hospital, participants ensure that patients have clinical follow-up and access to needed medications and support services:

The idea is to… make sure that they are able to function. They have the support that they need, and they’re cognitively fine…. They’re not having complications of the perioperative period. All the services they would need like physical therapy… they would need they should have. That is what our concern is. They have their medications, and their pain is controlled. So we make a phone call within 48 hours of their discharge, and that’s a must for our practice. (Participant 24)

Geriatricians also triage and manage patient postoperative questions, often reaching out to the surgical team for clarification:

They call us and [ask] does this suture look normal… and then I did… a video visit looking at things like Steri-Strips, I’m like, “I’m pretty sure from looking at this note that you’re just supposed to leave those in place, and I’ll send a picture to your oncologist, or your surgical oncologist, but I think your incision is fine but really I can’t tell them that definitively.” (Participant 18)

## Discussion

In this qualitative study of geriatricians, we presented novel findings on the actions geriatricians take to support older patients considering surgery. Navigating older patients through surgery is complex and requires that geriatricians provide support to patients and their families through a multipronged approach that has not been well delineated in the literature. Current guidelines comment on medical optimization, perioperative medical management, and the importance of advance care planning but fall short in describing all of the actions required from a primary care perspective to optimize care of older patients requiring surgery.^9,15^ Drawing on the expertise of geriatricians who navigate this process frequently, we present the first comprehensive framework that primary care clinicians may reference in this setting to tailor their practice to meet the needs of older patients.

It is important to note that this framework reflects the significant work that is performed by geriatricians and primary care clinicians to add value to the care of older and frail adults. This work ensures that surgical care is safe and effective as well as centered on patient goals. However, despite clear benefits to patients and the health care system, the identified work of geriatricians in this study is largely uncompensated and time intensive. Previous studies have shown that financial incentives for primary care clinicians targeting specific areas of primary care have led to improvements in health care quality.^16^ Therefore, we advocate for the development of incentive systems that will adequately reimburse primary care clinicians for doing this critical work.

A key finding that emerged from this work is the identification of multiple barriers encountered by geriatricians when supporting older adults undergoing surgical consultation and in the perioperative period. For example, geriatricians identify one of their key roles as eliciting and communicating patient goals so that they are well aligned with clinical care decisions. Geriatricians feel that the EMR is limited in capturing and conveying patient goals. These findings are supported by previous studies that have shown that primary care clinicians desire standardized workflows within the EMR for documenting advanced care planning discussions.^17^ This presents an area where health care systems could support primary care clinicians in caring for older patients.

Another area where geriatricians encounter barriers is in their communication with the surgical team in the preoperative and postoperative setting. Prior research has shown gaps in communication between primary care clinicians and surgical teams during the referral process and transitions of care.^18,19,20^ Evidence-based interventions that may improve these communication challenges include use of nurse care coordinators, adoption of health information technology, and ensuring that primary care clinicians have adequate time per patient visit.^20^

### Strengths and Limitations

A strength of this study is that the use of semistructured interviews allowed for an in-depth exploration of the lived experiences of geriatricians that is more detailed than what could have been obtained from a questionnaire. Further, our sample included a national sample of geriatricians from multiple practice types, including academic, community, private practice, Veterans Affairs, and Program of All-Inclusive Care for the Elderly programs, increasing the generalizability of the findings.

However, the study also had some limitations; the results may be less applicable to clinicians who practice in countries where these models of care are not applicable. This study did not investigate patient and caregiver perspectives, an area that may be of interest for future study.

## Conclusions

In this qualitative study of geriatricians, 7 key domains of perioperative care for older adults were identified. As the population ages, primary care clinicians will support older adults undergoing surgery with increasing frequency. We present the first comprehensive framework, to our knowledge, that primary care clinicians may reference when providing care for older adults throughout the surgical continuum.

## Notes

Supplement 1. **eAppendix.** Interview Guide

Supplement 2.
Data Sharing Statement
