# Supplementary material for: A Framework to Optimize Primary Care of Older Surgical Patients: A Qualitative Study of Geriatricians
Source: JAMA Netw Open. 2025 Jan 27;8(1):e2456787. doi: 10.1001/jamanetworkopen.2024.56787 (PMC11774092; doi:10.1001/jamanetworkopen.2024.56787)
Supplement: Supplement 1. — eAppendix. Interview Guide [file jamanetwopen-e2456787-s001.pdf]

Supplemental Online Content

Leonard ME, Williamson AJH, Weiss R, Kaphingst KA, Supiano MA, Cohan JN. A framework to optimize primary care of older surgical patients: a qualitative study of geriatricians. *JAMA Netw Open*. 2025;8(1):e2456787. doi:10.1001/jamanetworkopen.2024.56787

eAppendix. Interview Guide

This supplemental material has been provided by the authors to give readers additional information about their work.

© 2025 Leonard ME et al. *JAMA Network Open.*

eAppendix. Interview Guide

Study introduction:

- Thank you for participating, we very much appreciate your time. We will use this interview to learn about your perspectives on surgical consultations for your patients.
- For the following questions, please answer as though you are providing primary care as opposed to acting as a geriatric consultant.
- The typical patient in these scenarios is a moderately frail patient who is living at home, although we welcome for you to share your perspectives on patients across the frailty spectrum for any questions.
- For surgical conditions, you can envision more "elective" surgery, such as for a hernia that causes discomfort or recurrent, uncomplicated diverticulitis.
- I will turn on the recorder now. **Begin Recording.**
- Today's date is [xx]. This is [xx] interviewing participant [xx]. Do I have your consent to proceed with the interview and record our session?

Interview Questions:

1. What goes through your mind when your moderately frail patient develops a condition that may require surgery, such as a hernia or diverticulitis?
    a. How do you go about deciding whether to refer to a surgeon?
    b. Are there situations where you do not refer to a surgeon?
    c. In what ways do your patient's goals and preferences factor into your decision about referring?
    d. How do you go about eliciting your patient's goals and preferences about surgery?
2. What is your typical process for surgical referral?
3. After you make the decision to refer for surgery, how do you prepare your patient for the surgical consultation?
4. Once you have decided to refer, what is your goal for the surgical consultation?
5. In what ways are you typically involved in decision-making about whether or not your patient should have surgery?
    a. How would you ideally be involved?
    b. What would help you be involved in decision-making about surgery to the level you prefer?
    c. Are there ways in which you feel that decision-making about surgery could be improved?
6. In what ways do you typically interact with the consulting surgeon?
    a. How would you ideally interact?
    b. What would help you interact with the consulting surgeon to the level you prefer?
7. Do you see a role for a surgeon knowing a patient's goals and preferences about surgery?
    a. If yes, how do you envision surgeons learning about a patient's goals and preferences?
    b. If no, why not?

© 2025 Leonard ME et al. *JAMA Network Open.*

8. Could you tell me about a time when you felt uncomfortable referring a patient for surgical consultation?
9. Could you tell me about a time when you were unhappy with the outcome of a surgical consultation?
10. Once a decision about moving ahead with surgery is made, how do you typically follow your patient before surgery?
    a. How would you ideally be involved in the care of your patient before surgery?
    b. What would help you provide care to your patient before surgery?
11. How do you typically follow your patient postoperatively? For this question, postoperatively means in the hospital and after discharge.
    a. How would you ideally be involved in the care of your patient postoperatively?
    b. What would help you provide care to your patient postoperatively?
12. Do you have any other thoughts you would like to share regarding surgical consultations or surgical care for your frail patient population?

Stop recording.

[If has already completed survey] I again thank you for your time. As a sign of our appreciation, we will send you a gift card by email within the next week.

[If has not yet completed survey] I again thank you for your time. I will send you a link to complete a brief survey in RedCAP. Once that is complete, I will send you a gift card by email.

© 2025 Leonard ME et al. *JAMA Network Open.*
