# Supplementary material for: A Framework to Optimize Primary Care of Older Surgical Patients: A Qualitative Study of Geriatricians
Source: JAMA Netw Open. 2025 Jan 27;8(1):e2456787. doi: 10.1001/jamanetworkopen.2024.56787 (PMC11774092; doi:10.1001/jamanetworkopen.2024.56787)
Supplement: Supplement 2. — Data Sharing Statement [file jamanetwopen-e2456787-s002.pdf]

Data Sharing Statement

Leonard. A Framework to Optimize Primary Care of Older Surgical Patients. *JAMA Netw Open*. Published January 27, 2025. doi:10.1001/jamanetworkopen.2024.56787

Data
Data available: No
